# Metabolism of Gluconeogenic Substrates by an Intracellular Fungal Pathogen Circumvents Nutritional Limitations within Macrophages

**DOI:** 10.1128/mBio.02712-19

**Published:** 2020-04-07

**Authors:** Qian Shen, Stephanie C. Ray, Heather M. Evans, George S. Deepe, Chad A. Rappleye

**Affiliations:** a Department of Microbiology, The Ohio State University, Columbus, Ohio, USA; b Division of Infectious Diseases, University of Cincinnati College of Medicine, Cincinnati, Ohio, USA; Universidade de Sao Paulo

**Keywords:** *Histoplasma*, carbon metabolism, intracellular pathogens, macrophages, pathogenesis, phagosomes, virulence

## Abstract

Microbial pathogens exploit host nutrients to proliferate and cause disease. Intracellular pathogens, particularly those exclusively living in the phagosome such as Histoplasma capsulatum, must adapt and acquire nutrients within the nutrient-limited phagosomal environment. In this study, we investigated which host nutrients could be utilized by *Histoplasma* as carbon sources to proliferate within macrophages. *Histoplasma* yeasts can grow on hexoses and amino acids but not fatty acids as the carbon source *in vitro*. Transcriptional analysis and metabolism profiling showed that *Histoplasma* yeasts downregulate glycolysis and fatty acid utilization but upregulate gluconeogenesis within macrophages. Depletion of glycolysis or fatty acid utilization pathways does not prevent *Histoplasma* growth within macrophages or impair virulence *in vivo*. However, loss of function in Pck1, the enzyme catalyzing the first committed step of gluconeogenesis, impairs *Histoplasma* growth within macrophages and severely attenuates virulence *in vivo*, indicating that *Histoplasma* yeasts rely on catabolism of gluconeogenic substrates (e.g., amino acids) to proliferate within macrophages.

## Abstract (precis)

*Histoplasma* is a primary human fungal pathogen that survives and proliferates within host immune cells, particularly within the macrophage phagosome compartment. The phagosome compartment is a nutrient-limited environment, requiring *Histoplasma* yeasts to be able to assimilate available carbon sources within the phagosome to meet their nutritional needs. In this study, we showed that *Histoplasma* yeasts do not utilize fatty acids or hexoses for growth within macrophages. Instead, *Histoplasma* yeasts consume gluconeogenic substrates to proliferate in macrophages. These findings reveal the phagosome composition from a nutrient standpoint and highlight essential metabolic pathways that are required for a phagosomal pathogen to proliferate in this intracellular environment.

## INTRODUCTION

Successful microbial pathogens must acquire nutrients from their host environment. These necessary factors include major nutrients such as carbon, nitrogen, and sulfur for microbial growth and proliferation. In addition, microbes must scavenge micronutrients such as essential metals (e.g., iron, copper, and zinc [1]). The process of nutritional immunity (1, 2), whereby pathogen accessibility to essential nutrients is restricted by the host, highlights the battle between pathogen and host and illustrates how starvation of microbial pathogens can be an effective control strategy. The task of nutrient acquisition is a greater challenge for intracellular pathogens that reside in compartments within host cells that are more nutritionally limited than for pathogens that escape into a nutrient-rich cytosol or infect nutrient-rich sites such as the bloodstream or gastrointestinal tract.

Carbon compounds are particularly important as carbon is the major constituent of structural molecules necessary for cellular function, growth, and replication. In addition, the oxidation of carbon molecules generates energy to support cellular functions and enzymatic reactions. Since glucose is the major carbon and energy source for host cells, it is generally abundant in extracellular spaces and thus is a preferred carbon source for many microbial pathogens during infection. Many intracellular pathogens also have access to glucose or glucose derivatives. Listeria monocytogenes, which escapes the phagosome to reside within the host cytosol, uses abundant glucose-6-phosphate as one carbon source (3). *Salmonella* relies on metabolizing glucose to replicate in the specialized *Salmonella*-containing vacuole (SCV) of macrophages (4). The intracellular parasites Toxoplasma gondii and Leishmania major use catabolism of glucose and glucosamine, respectively, for proliferation within specialized parasitophorous vacuoles (5, 6).

While glucose is a preferred carbon source for many pathogens, some pathogens are present in glucose-deprived host environments during infection and thus must use alternative carbon sources that are available in those particular host niches. The macrophage pathogen Mycobacterium tuberculosis uses acetyl coenzyme A (acetyl-CoA), which is postulated to be derived from fatty acids (7–9), and gluconeogenesis to replicate in the macrophage phagosome, suggesting that glucose is not available in the *Mycobacterium*-containing phagosomal compartment (10, 11). Following gamma interferon (IFN-γ) activation of macrophages, M. tuberculosis uses cholesterol as the major carbon source to replicate and to sustain persistent infections (12). Cryptococcus neoformans also encounters glucose-deprived host environments during infection, as gluconeogenesis is required to establish successful systemic infections (13). The intracellular bacterium Legionella pneumophila resides within the *Legionella*-containing vacuole (LCV), in which the bacterium acquires host proteasomal degradation products (e.g., amino acids) for its nutritional needs (14). During different stages of infection of macrophages, Candida albicans upregulates fatty acid catabolism, the glyoxylate shunt, and gluconeogenesis as well as amino acid catabolism, indicating catabolism of nonglucose substrates (15, 16).

The primary human fungal pathogen Histoplasma capsulatum infects macrophages and proliferates within the phagosome. During infection, *Histoplasma* yeasts are found nearly exclusively within phagocytes, forcing the yeasts to adapt to the available resources within these host cells and specifically within the phagosome. For example, due to the absence of vitamins in the phagosome, intracellular *Histoplasma* yeasts must rely on *de novo* vitamin biosynthesis (17). In addition, intracellular yeasts upregulate factors for acquisition of metal ions (18–20), confirming that some nutrients are absent or scarce in the phagosome. It is unknown which carbon substrates are available to *Histoplasma* yeasts in the phagosomal environment and which are catabolized for *Histoplasma* yeast growth. To probe the nutrient composition of the *Histoplasma*-containing phagosome, we measured metabolic gene expression and tested the ability of mutants in metabolic pathways to proliferate within macrophages. The intracellular transcriptome of *Histoplasma* showed downregulation of glycolysis and fatty acid utilization but upregulation of gluconeogenesis, suggesting that hexoses and fatty acids do not serve as growth substrates for intracellular *Histoplasma* yeasts. Consistent with this, depletion of glycolysis-specific and fatty acid utilization enzymes did not impair *Histoplasma*’s virulence. However, loss of gluconeogenesis function attenuated *Histoplasma* virulence. Together with studies on catabolizable carbon molecules *in vitro*, these results suggest that *Histoplasma* relies on nonhexose, non-fatty acid gluconeogenic carbon substrates to meet its carbon requirements for growth within the phagosome. These findings provide the first indications of the carbon nutrient composition of the *Histoplasma*-containing phagosome and the metabolism that facilitates *Histoplasma* proliferation within macrophages.

## RESULTS

### *Histoplasma* yeasts catabolize hexoses and amino acids but not fatty acids.

**FIG 1:**
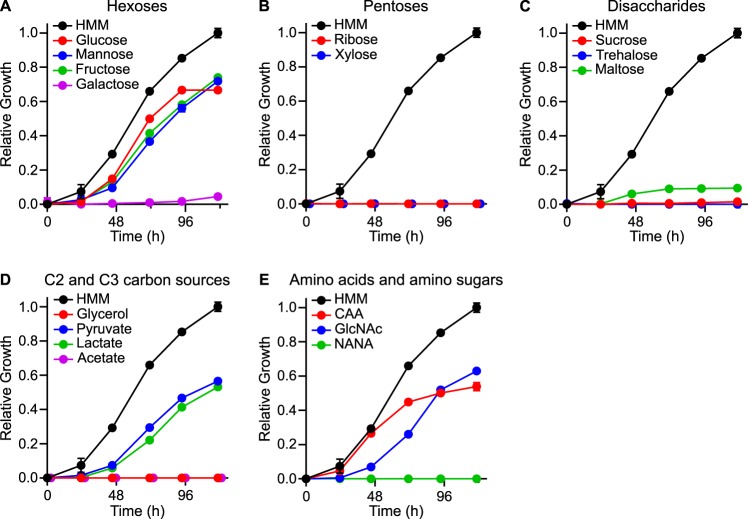
*Histoplasma* yeasts catabolize hexoses or amino acids as carbon sources. Data curves show *Histoplasma* yeast growth in liquid media containing different organic molecules as the carbon source. Yeasts were inoculated into a minimal medium containing 1.5% (wt/vol) (A) hexoses, (B) pentoses, (C) disaccharides, (D) C2 or C3 carbon substrates, or (E) amino sugars and amino acids as the carbon source. Yeast growth was measured as increasing turbidity (optical density [OD] at 595 nm) after incubation at 37°C. Growth was normalized to the maximal growth in rich medium (HMM; black symbols). Data represent the average relative growth levels ± standard deviations of results from biological replicates (*n* = 3).

To identify the carbon substrates that *Histoplasma* could potentially catabolize in the macrophage, we tested the ability of *Histoplasma* to grow *in vitro* on different organic compounds as the carbon source. To focus on carbon compound utilization, the growth media contained an inorganic nitrogen source (ammonium sulfate) and cysteine as a sulfur source since *Histoplasma* yeasts require organic sulfur (21). The concentration of cysteine was 25 μM, which was sufficient to meet the sulfur requirement but too low to supply carbon for growth and metabolism (see Fig. S1 in the supplemental material). Among the carbohydrates common to host biology, *Histoplasma* yeasts were restricted to growth on hexoses, including glucose, mannose, and fructose but not galactose (Fig. 1A). Neither pentoses (ribose or xylose; Fig. 1B) nor disaccharides (sucrose, trehalose or maltose; Fig. 1C) supported *Histoplasma* yeast growth. Interestingly, this limited utilization of carbohydrate substrates was specific to yeasts but not mycelia, since mycelia could grow on a broader range, including galactose, ribose, xylose, or maltose as the sole carbon source (Table 1). The failure to grow on sucrose, despite the ability of *Histoplasma* to grow on the two monosaccharides (glucose and fructose) that make up the disaccharide, likely results from its absence from the genome of the gene encoding Suc2 (sucrose-6-phosphate hydrolase). The *Histoplasma* genome does not encode homologs of trehalose transporters, and accordingly, *Histoplasma* could not utilize trehalose. For C2 or C3 carbon compounds, *Histoplasma* yeasts grew in media with pyruvate or lactate carbon sources but not glycerol or acetate (Fig. 1D) despite the presence of metabolic pathways for glycerol and acetate metabolism. *Histoplasma* yeasts grew well on amino acids (Casamino Acids [CAA]) (Fig. 1E). Among the amino sugars common to host glycans, *Histoplasma* yeast catabolized *N*-acetylglucosamine (GlcNAc) but not N-acetylneuraminic acid (NANA) as the carbon source (Fig. 1E). An additional NAm2 isolate (Hc01) as well as two North American type I (NAm1) class strains (WU24 and Hc17), a Panama strain (G186A), and a Latin America class strain (LAm; Hc30) all showed the same carbon source utilization (see Table S1 in the supplemental material), indicating that the carbon substrates metabolized by yeasts were not unique to the G217B NAm2 isolate. *Histoplasma* yeasts were unable to grow on free short-chain fatty acids, and short-chain fatty acids inhibited the growth of yeasts on glucose-containing media (Fig. S2). Attempts to grow *Histoplasma* yeasts on long-chain fatty acids were similarly unsuccessful (data not shown). These results show that *Histoplasma* yeasts catabolize a limited range of carbon molecules and focus attention on hexoses (including amino hexoses) and amino acids as potential carbon sources during infection.

**TABLE 1:** *Histoplasma* mycelium growth on different carbon substrates

Carbon substrate	Mycelial growth score^*a*^
Glucose	2
Fructose	2
Mannose	4
Galactose	3
Ribose	2
Xylose	2
Sucrose	0
Maltose	4
Trehalose	0
Glycerol	0
Pyruvate	0
Lactate	4
Acetate	0
Casamino Acids	2
N-Acetyl glucosamine	0
Bovine serum albumin	0

a Mycelial growth was scored visually using a 4-point qualitative scale as follows: a score of 0 represents no mycelial growth, and a score of 4 represents maximum mycelial growth.

10.1128/mBio.02712-19.1 FIG S1 *Histoplasma* yeasts cannot grow on cysteine as the sole carbon source. Data represent levels of *Histoplasma* yeast growth in liquid media containing different concentrations of cysteine as the sole carbon source. Yeasts were inoculated into a minimal medium containing a 22 μM, 88 μM, or 700 μM concentration of cysteine as the sole carbon source. Yeast growth was measured as increasing turbidity (optical density [OD] at 595 nm) after incubation at 37°C. Growth was normalized to the maximal growth in HMM (black symbols). Data represent average relative growth levels ± standard deviations of results from biological replicates (*n* = 3). Download FIG S1, EPS file, 0.1 MB. Copyright © 2020 Shen et al. 2020 Shen et al. This content is distributed under the terms of the Creative Commons Attribution 4.0 International license.

10.1128/mBio.02712-19.2 FIG S2 *Histoplasma* yeasts cannot catabolize short-chain fatty acids. Data curves show *Histoplasma* yeast growth in liquid media containing different short-chain fatty acids as the carbon source. Yeasts were inoculated into a minimal medium containing 0.2% (wt/vol) of formate, acetate, propionate, or butyrate as the carbon source without (A) or with (B) 1.5% glucose (wt/vol). Yeast growth was measured as increasing turbidity (OD at 595 nm) after incubation at 37°C. Growth was normalized to the maximal growth in glucose (black symbols). Data represent average relative growth levels ± standard deviations of results from biological replicates (*n* = 3). Download FIG S2, EPS file, 0.2 MB. Copyright © 2020 Shen et al. 2020 Shen et al. This content is distributed under the terms of the Creative Commons Attribution 4.0 International license.

10.1128/mBio.02712-19.5 TABLE S1 Yeast growth of phylogenetically distinct *Histoplasma* strains on carbon substrates. Yeast-phase cells of *Histoplasma* were tested for the ability to utilize the indicated substrates as the organic carbon source. Clinical isolates of *Histoplasma* were chosen to represent different phylogenetic groups as follows: Panama, North America type 1 (NAm1), North America type 2 (NAm2), and Latin America (LAm). Growth was scored using the following qualitative scale: +, good growth; +/−, weak growth and development of pseudohyphae; −, no growth. Download Table S1, PDF file, 0.1 MB. Copyright © 2020 Shen et al. 2020 Shen et al. This content is distributed under the terms of the Creative Commons Attribution 4.0 International license.

### *Histoplasma* yeasts can use digested protein, but not intact protein, as carbon sources.

**FIG 2:**
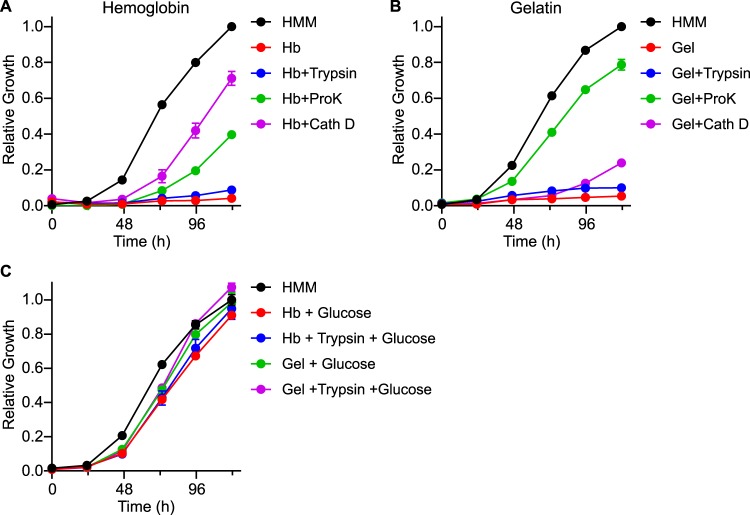
*Histoplasma* can use digested proteins, but not intact proteins, as the carbon source. Growth curves show *Histoplasma* yeast growth in minimal media containing 5 mg/ml of (A) human hemoglobin or (B) gelatin. For each, the protein was left intact (red symbols) or proteolytically digested with trypsin (0.2 μg/ml, blue symbols), proteinase K (50 μg/ml, green symbols), or cathepsin D (1.0 μg/ml, purple symbols) before inoculation with *Histoplasma* yeasts. (C) Growth curves show *Histoplasma* yeast growth in minimal media with 1.5% glucose in the presence of protein or proteolytically derived peptides. Growth was normalized to the maximal growth in rich medium (HMM; black symbols). Data represent average relative growth levels ± standard deviations of results from biological replicates (*n* = 3).

Since *Histoplasma* readily uses amino acids as a carbon source, we examined the potential to use host proteins to supply its carbon and energy needs during infection. Hemoglobin and gelatin were used as model proteinaceous growth substrates for *in vitro* growth in media lacking other carbon. *Histoplasma* yeasts were unable to grow in media with 5 mg/ml of protein unless the protein had previously been digested with proteinase K or the lysosomal proteinase cathepsin D to release amino acids and poly/oligopeptides (Fig. 2A and B). Cathepsin D digestion of gelatin permitted *Histoplasma* growth, although to a lesser extent than proteinase K digestion. However, digestion of either protein with trypsin did not enable any yeast growth. The lack of yeast growth on trypsin peptides was not due to any inhibitory activity of the peptides or of the enzymes used to digest the protein, as supplementation of the proteolytic digest with glucose restored the growth (Fig. 2C). The requirement for proteolytic digestion of protein substrates to enable *Histoplasma* yeast growth is consistent with findings showing that the G217B strain does not secrete proteases (22). The *in silico* profile of proteinase K digestion of hemoglobin or gelatin produces more single amino acids and short peptides than the *in silico* profile of trypsin digestion of these proteins. This suggests that *Histoplasma* utilization of proteolytic products is limited to single amino acids or short peptides.

### *Histoplasma* growth in macrophages upregulates gluconeogenesis.

**FIG 3:**
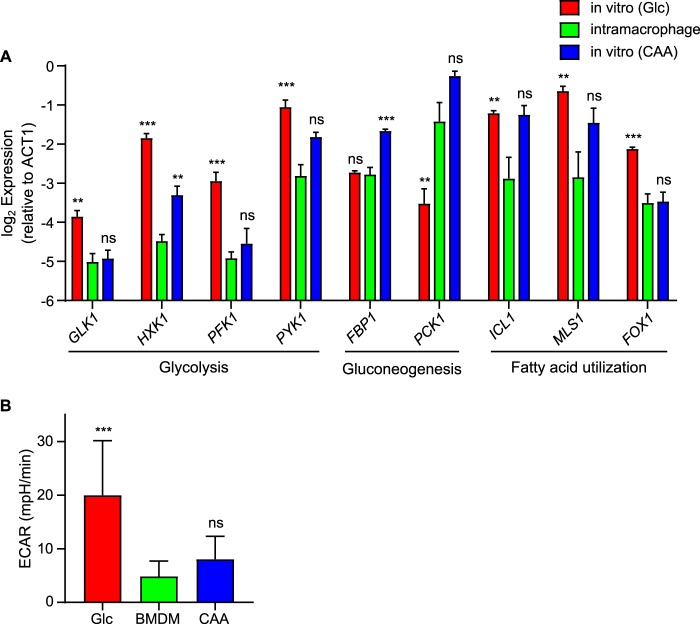
Intramacrophage growth downregulates *Histoplasma* glycolysis and fatty acid utilization but upregulates gluconeogenesis. (A) The transcription of genes involved in major carbon metabolism pathways (i.e., glycolysis, gluconeogenesis, and fatty acid utilization) was determined for *Histoplasma* yeasts grown *in vitro* in minimal medium with glucose (Glc; red) or Casamino Acids (CAA; blue) as the carbon source or for yeasts residing within P388D1 macrophages for 24 h (green). Transcript levels of each metabolic gene were quantified by qRT-PCR and calculated relative to *ACT1* transcript levels. The genes assayed included genes encoding the glycolysis enzymes glucose kinase (*GLK1*), hexokinase (*HXK1*), phosphofructokinase (*PFK1*), and pyruvate kinase (*PYK1*); the gluconeogenesis enzymes fructose-1,6-bisphosphatase (*FBP1*) and phosphoenolpyruvate carboxykinase (*PCK1*); and genes encoding proteins involved in fatty acid utilization steps represented by isocitrate lyase (*ICL1*), malate synthase (*MLS1*), and fatty acyl oxidase (*FOX1*). Data represent average expression levels ± standard deviations of results from biological replicates (*n* = 3). Asterisks represent significant differences in transcript levels between intramacrophage-grown yeasts and yeasts grown in glucose or Casamino Acids media (**, *P < *0.01; ***, *P < *0.001; ns, *P > *0.05) as determined by two-tailed Student's *t* test. (B) Basal glycolytic capacity measured by extracellular acidification rate (ECAR) was determined by Seahorse analysis for *Histoplasma* yeasts grown *in vitro* in minimal medium with glucose (red) or Casamino Acids (blue) as the carbon source or for yeasts residing within bone marrow-derived macrophages for 24 h (green). ECAR was measured by transfer of yeasts into glucose minimal medium. Total ECAR was measured before addition of glycolysis inhibitor 2-DG, and nonglycolytic ECAR was measured after addition of nonmetabolizable 2-deoxy-glucose. Data represent average rates ± standard deviations of results from biological replicates (*n* = 3). Asterisks represent significant differences (*P < *0.001) in ECAR between intramacrophage-grown yeasts and glucose-grown yeasts or Casamino Acids-grown yeasts using Kruskal-Wallis one-way ANOVA on ranks with Dunn’s pairwise multiple-comparison test.

As an indicator of the metabolic processes and the potential carbon substrate(s) used by *Histoplasma* for proliferation within macrophages, the transcriptional profile was determined for intramacrophage *Histoplasma* yeasts. *Histoplasma* yeasts were collected from macrophages at 24 h following infection, and the expression levels of genes representative of glycolysis, gluconeogenesis, and fatty acid utilization were examined and compared to those of genes of *Histoplasma* yeasts grown on glycolytic (glucose) or gluconeogenic (Casamino Acids) carbon substrates *in vitro*. Intramacrophage yeasts responded to the phagosomal environment by downregulating glycolysis (glucose kinase, *GLK1*; hexokinase, *HXK1*; phosphofructokinase, *PFK1*; pyruvate kinase, *PYK1*) (Fig. 3A). The transcription of genes encoding enzymes for utilization of fatty acids, which included β-oxidation of fatty acids (fatty acyl oxidase, *FOX1*) and genes encoding enzymes of the glyoxylate pathway (isocitrate lyase, *ICL1*; malate synthase, *MLS1*), did not increase to levels above baseline levels in intramacrophage yeasts and were reduced in comparison to growth *in vitro* with glucose (Fig. 3A). Baseline expression of fatty acid metabolism genes, even in media lacking fatty acids, likely reflects the requirement for metabolism of endogenous fatty acids. In contrast to glycolysis and fatty-acid metabolism genes, intramacrophage yeasts showed increased expression of the gene encoding the gluconeogenesis-specific enzyme phosphoenolpyruvate (PEP) carboxykinase (*PCK1*). Comparison of gene expression profiles to growth on known carbon substrates (glucose or Casamino Acids) showed that the transcriptome of intramacrophage yeasts mirrored aspects of that of yeasts grown on Casamino Acids (Fig. 3A), suggesting that amino acids rather than hexoses could be the primary carbon source catabolized by *Histoplasma* yeasts within macrophages.

As a validation of the metabolic profile of intramacrophage yeasts, we examined the glycolytic capacity of *Histoplasma* yeasts collected from primary bone marrow-derived macrophages (BMDMs). Intramacrophage *Histoplasma* yeasts were compared to *Histoplasma* yeasts grown on glycolytic (glucose) or gluconeogenic (Casamino Acids) carbon substrates *in vitro*. The glycolytic capacity of yeasts was determined by shifting yeasts to glucose-containing media and measuring the glycolysis-dependent extracellular acidification rate (ECAR) using a Seahorse XF24 analyzer. Casamino Acids-grown yeasts showed about 3-fold less ECAR than glucose-grown yeasts, indicating that yeasts grown in gluconeogenic substrates have a reduced capacity for glycolysis (Fig. 3B). Yeasts isolated from macrophages showed about 4-fold less ECAR than glucose-grown yeasts, a level similar to that seen with Casamino Acids-grown yeasts (Fig. 3B). These results demonstrate that intramacrophage yeasts have downregulated glycolysis pathways, consistent with the transcriptional profile (Fig. 3A) that indicates that *Histoplasma* yeasts grown in macrophages utilize gluconeogenic substrates.

### *Histoplasma* intramacrophage growth and virulence do not require glycolysis.

**FIG 4:**
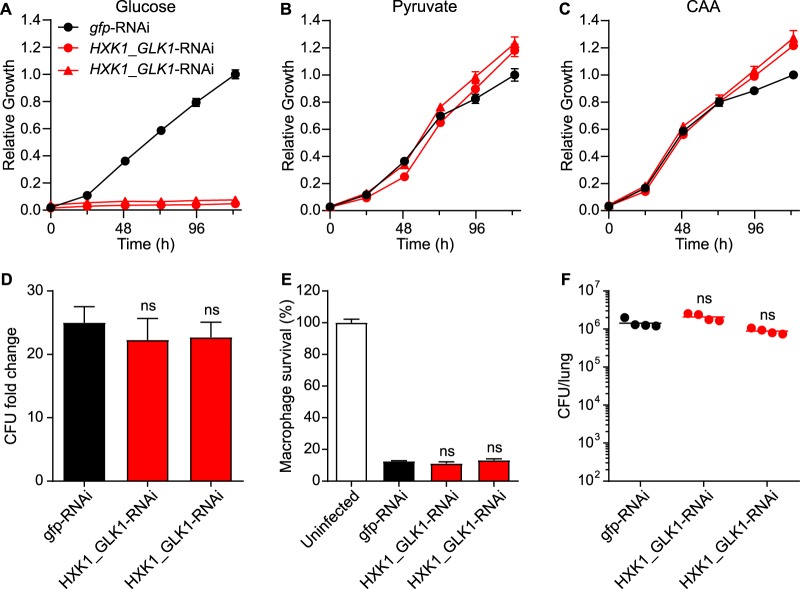
*Histoplasma* intramacrophage growth and virulence do not require hexose catabolism. RNAi was used to simultaneously deplete the hexokinase (*HXK1*) and glucose kinase (*GLK1*) genes that encode enzymes involved in the early steps of hexose catabolism. (A to C) Growth curves show the growth of Hxk1-expressing and Glk1-expressing yeasts (*gfp*-RNAi; black) or growth on two independent kinase-depleted lines (*HXK1*_*GLK1*-RNAi; red) in minimal media containing (A) glucose, (B) pyruvate, or (C) Casamino Acids (CAA) as the carbon source. Yeast growth was measured by determination of the optical density at 595 nm (OD_595_) and normalized to the maximal growth of kinase-expressing *Histoplasma* yeasts in each individual carbon substrate. Data represent average relative growth levels ± standard deviations of results from biological replicates (*n* = 3). (D and E) P388D1 macrophages were infected with Hxk1-expressing and Glk1-expressing or Hxk1-depleted and Glk1-depleted strains (MOI 1:2) and (D) the proliferation of intracellular yeasts and (E) the yeasts’ ability to lyse macrophages were determined. (D) Relative intracellular proliferation levels were determined by lysis of macrophages and comparison of the intracellular levels of viable yeasts (CFU) in the lysate at 0 h or 48 h postinfection. (E) The survival of P388D1 macrophages was quantified after 7 days of infection by measurement of the remaining macrophage-produced β-galactosidase activity. Data represent the average yeast growth levels or macrophage survival rates (compared to uninfected macrophages) ± standard deviations among replicate biological infections (*n* = 3). (F) *Histoplasma* Hxk1-expressing and Glk1-expressing or Hxk1-depleted and Glk1-depleted yeasts were used to establish sublethal respiratory infections in mice, and the fungal burdens in lungs were determined at 8 days postinfection by plating lung homogenates on solid medium and enumerating CFU. Data points represent the CFU from each mouse. Horizontal bars indicate the average lung fungal burdens (*n* = 4) with no significant (ns; *P > *0.05) differences between kinase-expressing and kinase-depleted strains as determined by two-tailed Student's *t* test.

Although *in vitro* growth tests indicated that *Histoplasma* can utilize various hexoses or GlcNAc for carbon needs (Fig. 1A and E), transcriptional profiling suggested that glycolytic substrates were not available or used by intraphagosomal yeasts. To provide functional validation of the intracellular metabolism suggested by the transcriptome profile of intramacrophage yeasts, the virulence of *Histoplasma* yeasts lacking the ability to catabolize hexoses was tested. We used a chimeric RNA interference (RNAi) trigger to simultaneously deplete hexokinase (Hxk1) and glucokinase (Glk1), which are required for the first step in hexose catabolism. The Hxk1- and Glk1-depleted yeasts could not use glucose (Fig. 4A) but could grow on pyruvate and Casamino Acids (Fig. 4B and C), showing the effectiveness of the RNAi-based depletion in specifically blocking hexose catabolism. Preventing utilization of hexoses did not impair proliferation of *Histoplasma* within macrophages (Fig. 4D), and this growth enabled normal killing of the host macrophages (Fig. 4E). Loss of hexose catabolism similarly did not attenuate *Histoplasma* virulence *in vivo* as fungal burdens in the lungs of infected mice were equal to those of mice infected with glycolysis-competent *Histoplasma* yeasts (Fig. 4F). These data support the transcriptional analysis results and demonstrate that hexose catabolism is not necessary for *Histoplasma* yeasts to proliferate in macrophages.

### *Histoplasma* intramacrophage growth and full virulence require gluconeogenesis.

**FIG 5:**
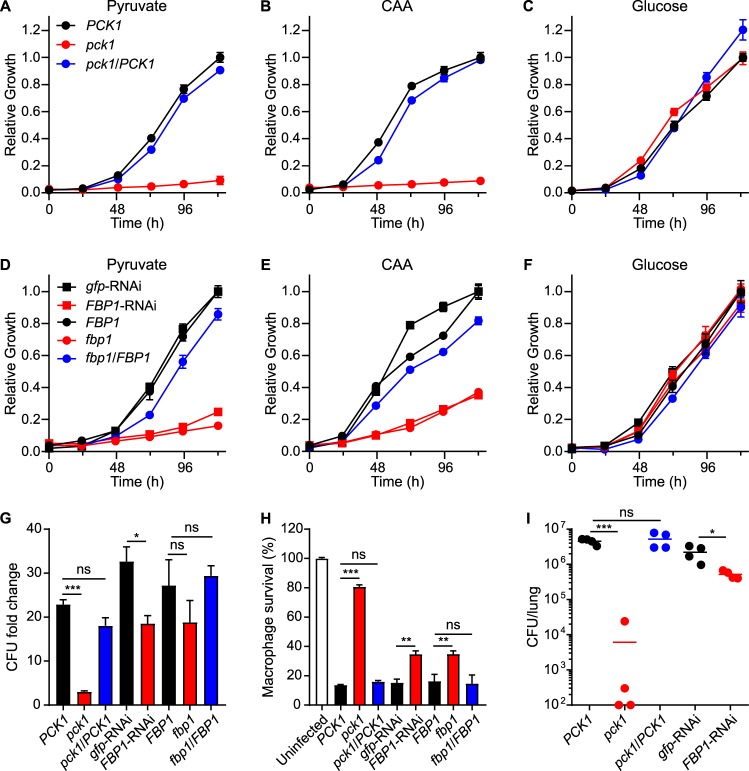
*Histoplasma* intramacrophage growth and virulence require gluconeogenesis. (A to C) Growth curves indicate the *in vitro* growth levels of Pck1-expressing (*PCK1*; black), Pck1-deficient (*pck1*; red), and *PCK1*-complemented mutant (*pck1*/*PCK1*; blue) *Histoplasma* yeasts in minimal media containing (A) pyruvate, (B) Casamino Acids (CAA), or (C) glucose as the carbon source. (D to F) Growth curves show the *in vitro* growth levels of Fbp1-expressing (*gfp*-RNAi or *FBP1*; black square or black circle, respectively), Fbp1-depleted (*FBP1*-RNAi; red square), loss of Fbp1 (*fbp1*; red circle), and Fbp1-complemented (*fbp1*/*FBP1*; blue circle) strains in minimal media containing (D) pyruvate, (E) Casamino Acids, or (F) glucose as the carbon source. Growth was measured by OD_595_ and normalized to the maximal growth of Fbp1-expressing yeasts. Data represent average relative growth levels ± standard deviations of results from biological replicates (*n* = 3). (G and H) P388D1 macrophages were infected with *Histoplasma* yeasts (MOI 1:2), and the proliferation of yeasts within macrophages (G) and the ability of yeasts to lyse macrophages (H) were determined. (G) The relative intracellular proliferation rates were determined by lysis of macrophages and comparison of the intracellular levels of viable yeasts (CFU) in the lysate at 0 h or 48 h postinfection. (H) The survival of P388D1 macrophages was quantified after 7 days of infection by measurement of the remaining macrophage-produced β-galactosidase activity. Data represent the average yeast growth levels or macrophage survival rates (compared to uninfected macrophages) ± standard deviations among biological replicate infections (*n* = 3). (I) Gluconeogenesis-competent (*PCK1* or *gfp*-RNAi), Pck1-deficient (*pck1*), or Fbp1-depleted (*FBP1*-RNAi) *Histoplasma* yeasts were used to inoculate mice intranasally, and the fungal burdens in lungs were determined at 8 days postinfection by plating lung homogenates on solid medium, to enumerate CFU. Data points represent CFU from each mouse, and horizontal bars indicate the average lung fungal burdens. Asterisks indicate significant (*, *P < *0.05; **, *P < *0.01; ***, *P < *0.01) differences compared to either the Pck1- or Fbp1-expressing controls as determined by two-tailed Student's *t* test.

Consistent with the intramacrophage transcriptional shift to gluconeogenesis, intramacrophage growth of *Histoplasma* requires the gluconeogenic enzyme Pck1. Through a forward genetic screen for yeasts unable to grow within macrophages (20), we isolated mutants with significantly reduced proliferation in macrophages which included three insertional mutants with disruptions in the *PCK1* locus (Fig. S3). Loss of Pck1 function, which catalyzes the first committed step of gluconeogenesis, deprived yeasts of the ability to grow on gluconeogenic substrates (pyruvate and Casamino Acids [Fig. 5A and B, respectively]) but did not impair growth on glucose (Fig. 5C). Introduction of the wild-type *PCK1* gene into the *pck1* mutant rescued growth on gluconeogenic substrates (Fig. 5A and B). In addition to the *pck1* mutants, we depleted a second gluconeogenesis-specific enzyme, fructose-1,6-bisphosphatase (Fpb1), by the use of RNAi to test its function. Depletion of Fbp1 reduced, but did not eliminate, yeast growth on gluconeogenic substrates (pyruvate and Casamino Acids [Fig. 5D and E, respectively]) but did not impair growth on a glycolytic substrate (glucose [Fig. 5F]).

10.1128/mBio.02712-19.3 FIG S3 Schematic representation of T-DNA insertions in the *PCK1* locus. Three independent *pck1* mutants (OSU151, OSU304, and OSU305) were isolated from the genetic screen for mutants impaired in intramacrophage growth. The orientation of the T-DNA insertion (yellow) is depicted by its left border (LB) and right border (RB). In OSU151, the T-DNA was inserted 90 bp upstream of the start codon (ATG). In OSU304, the T-DNA was inserted into the intron 283 bp downstream of the third exon. In OSU305, only the right border of the T-DNA was mapped, which localized the T-DNA element at 27 bp from the start of the fourth exon. Download FIG S3, EPS file, 0.03 MB. Copyright © 2020 Shen et al. 2020 Shen et al. This content is distributed under the terms of the Creative Commons Attribution 4.0 International license.

In contrast to loss of glycolysis, prevention of gluconeogenesis reduced intramacrophage growth and virulence of *Histoplasma*. Loss of Pck1 function severely impaired proliferation of *Histoplasma* yeasts within macrophages (Fig. 5G), and the reduced proliferation prevented *Histoplasma* lysis of macrophages (Fig. 5H). Depletion of Fbp1 reduced intramacrophage growth compared to that seen with Fbp1-expressing yeasts (Fig. 5G), which attenuated the ability of Fbp1-depleted yeasts to eventually lyse host macrophages (Fig. 5H). Following infection of mice, loss of Pck1 or Fbp1 function reduced lung fungal burdens (Fig. 5I), with loss of Pck1 causing a stronger reduction than depletion of Fbp1 (Fig. 5I). Since depletion of Fbp1 attenuated virulence less than loss of Pck1, we examined the degree of elimination of Fbp1 activity. Quantification of enzymatic activity showed that roughly 3% of the activity remained for conversion of fructose-1,6-bisphosphate to fructose-6-phosphate in the Fbp1-depleted RNAi lines compared to wild-type yeasts (Fig. S4A). Interestingly, this residual activity was similar to that of an *fbp1* loss-of-function mutant (Fig. S4A) which does not produce a functional Fbp1 enzyme. This suggests that other enzymes besides Fbp1 are present in *Histoplasma* that can dephosphorylate fructose 1,6-bisphosphate, resulting in the attenuation of Fbp1-deficient yeasts being less pronounced than that of Pck1-deficient yeasts. Nonetheless, both functional tests showed that a reduced capacity to catabolize gluconeogenic substrates attenuates *Histoplasma* virulence, indicating that *Histoplasma* yeast must catabolize gluconeogenic substrates during infection of macrophages.

10.1128/mBio.02712-19.4 FIG S4 Depletion of Fbp1 and Icl1 enzymatic activity in *Histoplasma* RNAi and mutant lines. (A) Fructose-1,6-bisphosphatase activity was determined in lysates from Fbp1-expressing (*FBP1*) and Fbp1-deficient (the *fbp1* frameshift mutant and *FBP1*-RNAi) *Histoplasma* yeasts. Activity was determined by the measurement of NADPH production (absorbance at 365 nm) using an enzyme-coupled assay (i.e., phosphoglucose isomerase and glucose-6-phosphate dehydrogenase) after addition of fructose-1,6-bisphosphate and NADP^+^. (B) The isocitrate lyase activity was determined by analysis of glyoxylate production in lysates from wild-type *Histoplasma* (*gfp*-RNAi) and two independent Icl1-depleted (*ICL1*-RNAi) *Histoplasma* strains. Activity was determined by the measurement of glyoxylate production as its phenylhydrazone derivative (quantified by absorbance at 324 nm) after addition of isocitrate. For both assays, cellular lysates were prepared from yeasts grown in HMM to the late log phase and protein concentrations were determined by Bradford assay. Data represent average specific activity levels ± standard deviations of results from biological replicates (*n* = 3). Asterisks indicate significant (***, *P < *0.01) differences compared to the wild type as determined by two-tailed Student’s *t*-test. Download FIG S4, EPS file, 0.1 MB. Copyright © 2020 Shen et al. 2020 Shen et al. This content is distributed under the terms of the Creative Commons Attribution 4.0 International license.

**FIG 6:**
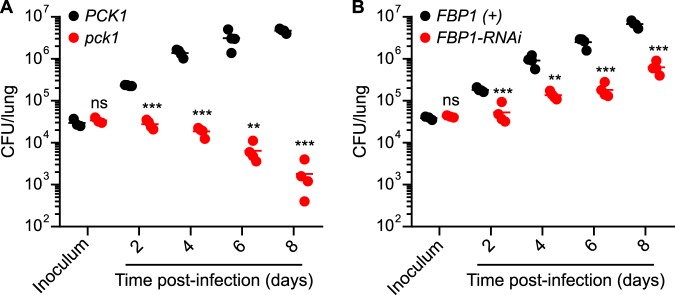
Attenuation of gluconeogenesis-deficient *Histoplasma* yeasts occurs during early infection. *Histoplasma* strains lacking the gluconeogenesis-specific Pck1 (A) or Fbp1 (B) functions were mixed equally with Pck1-expressing (*PCK1*) or Fbp1-expressing [*FBP1*(*+*)] yeasts and used to establish respiratory infections in mice. Lung fungal burdens were determined at 2, 4, 6, and 8 days postinfection by plating lung homogenates on solid medium to enumerate CFU. Individual strains were identified from the mixed pool of colonies by marking one of the strains of the mixed infection with fluorescence from a fluorescent protein transgene (*PCK1* [nonfluorescent] versus *pck1* [GFP fluorescent] or *FBP1* [red fluorescent] versus *FBP1*-RNAi [nonfluorescent]). Data points represent CFU at each time point from individual mice, with horizontal bars indicating the average lung fungal burdens (*n* = 4 mice). Asterisks indicate significant (*, *P < *0.05; **, *P < *0.01) or nonsignificant (ns; *P > *0.05) differences between gluconeogenesis-deficient strains and the corresponding gluconeogenesis-competent strains as determined by two-tailed Student's *t* test.

To define when gluconeogenesis is required for *Histoplasma* infection of mice, we examined fungal burden kinetics over 8 days of infection. Wild-type yeasts and gluconeogenesis-deficient yeasts were differentially marked by expression of fluorescent protein (FP) to enable coinfection of mice and separate enumeration of each strain. Specifically, Pck1-deficient yeasts expressed green fluorescent protein (GFP) and were inoculated with GFP-negative Pck1-expressing yeasts. Since *FPB1*-RNAi used silencing of the sentinel *gfp* fluorescence (23) to indicate RNAi-based depletion of target, for the *FBP1*-RNAi-expressing and Fbp1-expressing coinfection, Fbp1-expressing yeasts were transformed with a construct for expression of red fluorescent protein (RFP). Quantification of the fungal burdens over 8 days showed that Pck1 deficiency or Fbp1 depletion reduced fungal burdens at the earliest time point (2 days postinfection; Fig. 6A and B). For Pck1-deficient yeasts, fungal burdens steadily declined from the inoculum, consistent with a requirement for gluconeogenesis throughout infection (Fig. 6A). Depletion of Fbp1 caused a reduced rate of fungal proliferation compared to wild-type yeasts (Fig. 6B). In both cases, the fungal burdens of wild-type yeasts continued to steadily increase over time, indicating that gluconeogenesis is required from the beginning and throughout infection. Together with the Hxk1 and Glk1 depletion results (Fig. 4F), these results indicate that gluconeogenic substrates, but not hexoses, support the intracellular proliferation of *Histoplasma* yeasts throughout the acute infection stage.

### *Histoplasma* intramacrophage growth and virulence do not require fatty acid utilization.

**FIG 7:**
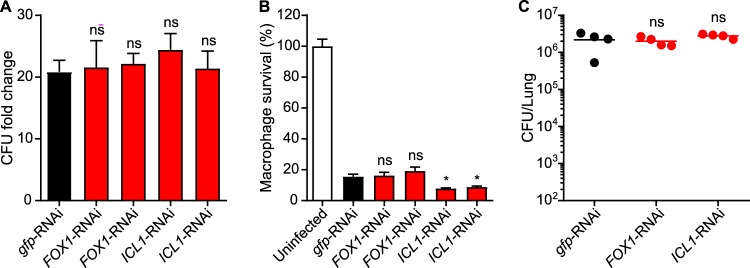
*Histoplasma* intramacrophage growth and virulence do not require fatty acid catabolism. Macrophages were infected with metabolically normal yeasts (*gfp*-RNAi) or two independent lines of Fox1-deficient or Icl1-deficient *Histoplasma* yeasts (MOI 1:2), and the proliferation of intracellular *Histoplasma* yeasts (A) or the yeasts’ ability to lyse macrophages (B) was determined. (A) The relative intracellular proliferation rates were determined by lysis of macrophages and comparison of the intracellular levels of viable yeasts (CFU) in the lysate at 0 h or 48 h postinfection. Macrophage survival (B) was quantified after 7 days of infection by measurement of the remaining macrophage-produced β-galactosidase activity. Data represent averages ± standard deviations of results from biological replicates (*n* = 3). (C) Mice were infected intranasally with metabolically normal, Fox1-depleted, or Icl1-depleted yeasts to establish respiratory disease. Fungal burdens were determined after 8 days by plating lung homogenates on solid medium and enumerating CFU. Data points represent the lung fungal burdens (CFU) in individual mice, and horizontal bars represent average fungal burdens. No significant differences (ns; *P > *0.05) between the strains were detected as determined by two-tailed Student's *t* test.

Although *Histoplasma* yeasts cannot grow on fatty acids *in vitro* (Fig. S2), as the primary component of host cell membranes, fatty acids are an abundant potential carbon substrate in hosts and incorporation of fatty acid-derived carbon into cellular molecules requires gluconeogenesis. To directly test whether catabolism of fatty acids contributes to *Histoplasma* virulence, we depleted two key enzymes (Fox1 and Icl1) required for β-oxidation of acyl chains and incorporation of the generated acetyl-CoA into central carbon metabolism, respectively. Since *Histoplasma* yeasts cannot grow on fatty acids or acetate *in vitro*, we confirmed the Icl1 depletion using an enzymatic assay, which showed that the RNAi effect reduced Icl1 activity to less than 5% (Fig. S4B). Preventing catabolism of fatty acids did not reduce intramacrophage proliferation of *Histoplasma* (Fig. 7A) and did not impair the ability to kill host macrophage cells (Fig. 7B). Both the Fox1-deficient and Icl1-deficient yeasts had lung fungal burdens similar to those seen with the wild type following infection of mice (Fig. 7C). Thus, Fox1-deficient and Icl1-deficient yeasts are fully virulent, indicating that *Histoplasma* does not use fatty acids as carbon sources for proliferation within macrophages.

### The virulence requirement for pyruvate synthesis indicates that pyruvate and alanine are unavailable to *Histoplasma* within the phagosome.

**FIG 8:**
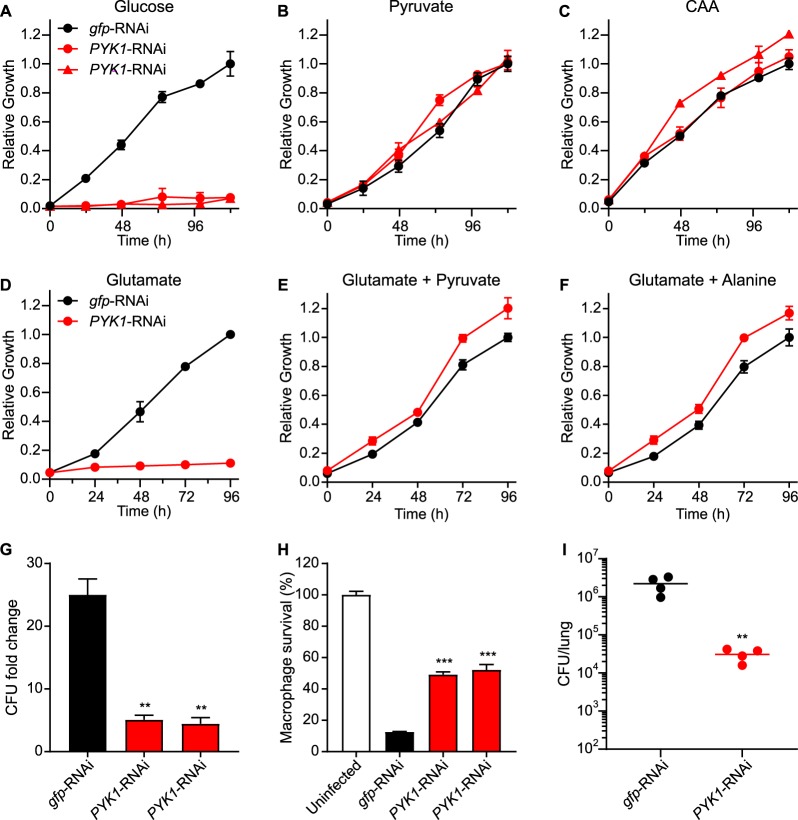
Pyruvate auxotrophy indicates lack of pyruvate and alanine availability during infection. (A to F) Growth curves show the *in vitro* growth of Pyk1-expressing (*gfp*-RNAi) or Pyk1-depleted (*PYK1*-RNAi) yeasts in minimal media containing (A) glucose, (B) pyruvate, (C) Casamino Acids, (D) glutamate, or glutamate supplemented with (E) pyruvate or (F) alanine as the carbon source(s). Yeast growth was measured by OD_595_ and normalized to the maximal growth of Pyk1-expressing *Histoplasma* yeasts in each carbon substrate. Data represent average relative growth levels ± standard deviations of results from biological replicates (*n* = 3). (G and H) Macrophages were infected with *Histoplasma* yeasts (MOI 1:2), and the proliferation of yeasts within macrophages (G) and the ability of yeasts to lyse macrophages (H) were determined. (G) The relative intracellular proliferation rates were determined by lysis of macrophages and comparison of the intracellular levels of viable yeasts (CFU) in the lysate at 0 h or 48 h postinfection. (H) Macrophage survival was quantified after 7 days of infection by measurement of the remaining macrophage-produced β-galactosidase activity. Data represent the average yeast growth levels or macrophage survival rates (compared to uninfected macrophages) ± standard deviations among biological replicate infections (*n* = 3). (I) Pyk1-expressing or Pyk1-depleted yeasts were used to inoculate mice intranasally, and the fungal burdens in lungs were determined at 8 days postinfection by plating lung homogenates on solid medium to enumerate CFU. Data points represent CFU from each mouse, and horizontal bars indicate the average lung fungal burdens. Asterisks indicate significant (**, *P < *0.01; ***, *P < *0.01) differences between the Pyk1-expressing and the Pyk1-depleted strains as determined by two-tailed Student's *t* test.

Both glycolysis and gluconeogenesis rely on pyruvate kinase (Pyk1) to create pyruvate, which is a precursor for alanine biosynthesis. To test the intramacrophage requirement for metabolism of carbon to pyruvate, we depleted Pyk1 function by RNAi. Pyk1-depleted yeasts were unable to grow on glucose as the carbon source (Fig. 8A) but were able to grow on pyruvate (Fig. 8B) or Casamino Acids (Fig. 8C) due to the conversion of alanine or serine to pyruvate. Yeast growth on an individual gluconeogenic amino acid, glutamate, is prevented by depletion of Pyk1 (Fig. 8D) but can be restored if pyruvate is added (Fig. 8E), indicating that conversion of gluconeogenesis-derived phosphoenolpyruvate to pyruvate is required for yeast growth. Yeast growth on glutamate as the carbon source is similarly restored if alanine is supplied (Fig. 8F), since alanine can be metabolized to pyruvate. Depletion of Pyk1 function prevented yeast proliferation within macrophages (Fig. 8G), which impaired the ability of yeasts to kill host macrophages (Fig. 8H). Without Pyk1 function, *Histoplasma* yeasts were also attenuated in lung infection as shown by reduced fungal burdens when mice were infected with Pyk1-depleted *Histoplasma* (Fig. 8I). Since either pyruvate or alanine can restore the growth of Pyk1-deficient yeasts with gluconeogenic carbon substrates *in vitro*, but intramacrophage proliferation of Pyk1-deficient yeasts remains impaired, these results indicate that pyruvate and alanine are not sufficiently available within the *Histoplasma*-containing phagosome to support yeast growth.

## DISCUSSION

In this report, we present multiple lines of experimental data that define the metabolism of *Histoplasma* residing within host macrophages. Prior studies of diverse pathogens that can infect macrophages suggested that intracellular pathogens catabolize host lipids (7, 15, 24). Two prominent examples are the findings that M. tuberculosis and C. albicans require enzymes of the glyoxylate and fatty acid β-oxidation pathways for full virulence *in vivo* (7–9, 15, 25). The finding that loss of Icl1 does not impair the virulence of Aspergillus fumigatus (26), an extracellular pathogen, further suggested that host fatty acid catabolism represented the carbon acquisition strategy of intracellular pathogens. However, *Histoplasma* yeasts cannot grow on fatty acids as the carbon source *in vitro*. In addition, intracellular *Histoplasma* yeasts downregulate fatty acid utilization pathways (i.e., Fox1 and Icl1; Fig. 3A). Consistent with the expression studies, preventing fatty acid utilization did not impair *Histoplasma* proliferation in macrophages and did not attenuate *Histoplasma* virulence in mice (Fig. 7). This difference may indicate that fatty acids are not available in the *Histoplasma*-containing phagosome (unlike that of other phagocytosed pathogens), that *Histoplasma* prefers to exploit different carbon sources in the phagosome, or that *Histoplasma* is simply unable to metabolize exogenous fatty acids.

As a nearly exclusive intracellular pathogen (27), *Histoplasma*’s requirement for certain metabolic pathways for intracellular growth provides insights into the available carbon substrates in the phagosomal environment. Our data show that during infection of macrophages, only the *Histoplasma* yeasts that were deficient in gluconeogenesis, but not in hexose catabolism, had impaired intracellular growth (Fig. 4 and 5). Since *Histoplasma* can utilize hexoses when they are available, this indicates that hexoses and modified hexoses (e.g., GlcNAc) are not sufficiently available in the phagosomal environment to support *Histoplasma* proliferation. This intracellular metabolism translates to attenuated virulence for mutants deficient in gluconeogenesis but not in hexose catabolism. This narrow metabolism range of intracellular *Histoplasma* yeasts contrasts with the highly plastic metabolism of other fungal pathogens that have both intracellular and extracellular phases during host infection. For example, C. albicans encounters diverse host environments (e.g., gastrointestinal tract, blood, and kidney), each with different carbon molecules, which requires this pathogen to rely on multiple metabolic pathways. Indeed, C. albicans virulence *in vivo* requires Icl1, Pck1, Fbp1, and Pyk1, all of which are involved in competing carbon metabolism pathways (25, 28–30), reflecting the different forms of nutrient metabolism associated with different intracellular and extracellular environments in the host and explaining why infections with cultured macrophages often do not translate into the same *in vivo* phenotypes. Similarly to that of C. albicans, full virulence of C. neoformans also relies on both glycolysis and gluconeogenesis, depending on the infection model; C. neoformans’s virulence requires Pck1 in a murine tail vein infection model, whereas Hxk1/Hxk2 and Pyk1 are required for virulence in a murine inhalation model (13, 31). In addition, C. neoformans has a significant extracellular life where it may encounter carbon substrates that differ from those in the phagosome.

Combining the intracellular *Histoplasma* growth requirement for gluconeogenesis and the limited spectrum of substrates that *Histoplasma* can metabolize suggests that *Histoplasma* growth within the phagosome employs protein or amino acid catabolism. Glucose serves as the main carbon source for host tissues; consequently, cell culture medium is typically a rich medium dominated by glucose. The *in vitro* growth medium for most fungal pathogens, including that used for antifungal susceptibility testing (32), is modeled after this, being rich in diverse carbon substrates, particularly glucose. There is growing recognition that such *in vitro* growth media can be quite dissimilar from the actual host environments encountered by the pathogen. For example, *Candida* vulvovaginitis is more appropriately modeled with lactate as a carbon source, under which conditions *Candida* cells have a different cell wall composition (33, 34). Our transcriptional and Seahorse analysis, combined with the data showing the full virulence of hexose catabolism-deficient mutants, indicates that a medium centered on glucose is not reflective of *Histoplasma* yeast metabolism during infection. Exploration of gluconeogenic carbon molecules potentially found in the host showed that *Histoplasma* used amino acids efficiently (Fig. 1E), suggesting that amino acids may serve as the principal carbon substrate in the *Histoplasma*-containing phagosome. A precedent for amino acid utilization by pathogens within the phagosome exists; *Legionella* uses host amino acids derived from proteasome proteolysis to support growth in the phagosome (14), and C. albicans can use amino acid metabolism to generate ammonia, thereby alkalinizing the environment to signal the transition to hyphal growth (16, 35). Interestingly, *Histoplasma* was able to grow on protein as the carbon substrate only under conditions in which the protein was sufficiently digested into oligopeptides or individual amino acids (Fig. 2A and B). However, not all peptides/amino acids supported growth of *Histoplasma* yeasts; tryptic peptides could not supply the carbon required for yeast growth, but cathepsin D-digested protein could. This suggests that the intraphagosomal catabolism of *Histoplasma* is limited to certain amino acids or specific peptide compositions. As cathepsin D is present in the phagolysosome, cathepsin D proteolysis of host substrates may be one avenue by which *Histoplasma* yeasts obtain carbon molecules for growth within the phagosome. Alternatively, *Histoplasma* yeasts may secrete proteinases while resident within macrophages for the same purpose. However, only North American type I (NAm1) strains (and not the type II [NAm 2] strains used in this study) produce extracellular proteinase activity, at least *in vitro* (36).

Pyk1-deficient *Histoplasma* yeasts are unable to grow on gluconeogenic amino acids due to an inability to produce pyruvate from phosphoenolpyruvate (PEP), a requirement that can be met with catabolism of alanine. The inability of Pyk1-deficient yeasts to grow in macrophages thus suggests that alanine and pyruvate are unavailable in the *Histoplasma*-containing phagosome. Unlike *Histoplasma*, Pyk1-deficient *Saccharomyces* still can grow on ethanol as the sole carbon source due to the presence of the Mae1 malic enzyme, which converts malate into pyruvate (37). A search for Mae1-encoding genes in the *Histoplasma* genome identified a Mae1 ortholog (data not shown). However, the inability of Pyk1-deficient yeasts to grow on gluconeogenic amino acids suggests that pyruvate in *Histoplasma* yeast can be produced from PEP only through the activity of Pyk1 despite the presence of Mae1. Together with the growth on select proteolytic fragments, these data suggest that *Histoplasma* grows on a limited spectrum of peptides/amino acids that are present in the macrophage phagosome.

Depletion of the gluconeogenesis-specific Fbp1 enzyme did not attenuate *Histoplasma* virulence as much as loss of Pck1 (Fig. 5G to I). This was paralleled by an incomplete block of growth of Fbp1-depleted yeasts on gluconeogenic carbon substrates (Fig. 5D and E). One possible explanation is insufficient depletion of FBP1 expression by RNAi. However, the RNAi *gfp* sentinel indicated good silencing of *FBP1* expression. We have had a very successful history of depleting enzyme functions using RNAi (17, 38–40), and our RNAi-based depletion lines in this study successfully depleted the sentinel *gfp* to similar levels. Therefore, it is unlikely that our RNAi did not sufficiently deplete Fbp1 expression. Enzymatic assays showed that lysates from both *FBP1*-RNAi yeasts and an *fbp1* genetic mutant still had about 3% to 4% of the capability to dephosphorylate fructose-1,6-bisphosphate (see Fig. S4A in the supplemental material), an activity that is lost upon heat inactivation of total proteins in the lysate. This suggests that other enzymes in *Histoplasma* can dephosphorylate fructose 1,6-bisphosphate and provide a low level of the Fbp1-independent activity required for gluconeogenesis, explaining why the loss of Fbp1 function did not attenuate *Histoplasma* virulence to the same degree as loss of Pck1. In support of the idea of Fbp1-independent fructose-1,6-bisphosphatase activity, Aspergillus nidulans mutants with a gene knockout of *FBP1* also still grow on gluconeogenic carbon substrates (glutamate and ethanol) as the sole carbon source (41) and *FBP1* loss in Yarrowia lipolytica does not abolish its ability to grow on ethanol (42).

Despite the interconnectedness of metabolic pathways, taken together, our data indicate that *Histoplasma* growth within the macrophage phagosome involves catabolism of a narrow range of gluconeogenic substrates. Deficiency in the utilization of host carbon molecules may reduce the energy production of yeasts and/or biosynthesis of carbon-containing molecules for cell structure and function. Even a slight reduction in the formation of metabolic intermediates, while limiting fungal growth, may also impair the ability of yeasts to produce virulence-enhancing determinants such as the alpha-(1,3)-glucan cell wall layer (43) or enzymes to combat macrophage defenses (44, 45) or modulate the pH of the *Histoplasma*-containing phagosome (46, 47). Whether for metabolism and growth or for virulence factor production, acquisition and metabolism of host carbon molecules within the phagosome are pivotal to *Histoplasma* pathogenesis. Transcriptional analyses suggest, and results obtained with hexose and fatty acid catabolism-deficient strains confirm that intraphagosomal growth does not involve catabolism of hexose or fatty acid substrates (Fig. 4 and 7). Rather, the data indicate that *Histoplasma* utilizes a gluconeogenic substrate, likely amino acids or peptides found in the phagosomal compartment, which may be dependent on host proteolytic processes. *Histoplasma* mycelia, in contrast to the pathogenic yeasts, function as saprobes in the environment and are more flexible with respect to the carbon substrates that they can metabolize. This suggests that *Histoplasma* yeasts have adapted to live within the macrophage phagosome, specializing in catabolism of the gluconeogenic substrates that are available within this intracellular compartment.

## MATERIALS AND METHODS

### *Histoplasma* strains and growth.

The *Histoplasma* strains used in this study are listed in Table S2 in the supplemental material. North American clade 2 clinical isolate G217B (ATCC 26032) and strains derived from it were used throughout this study unless otherwise noted. For general maintenance of strains, *Histoplasma* yeasts were grown in *Histoplasma*-macrophage medium (HMM) or 3M medium (48) with Casamino Acids as the nonglycolytic carbon source for strains deficient in glycolysis. For growth of uracil auxotrophs, HMM was supplemented with 100 μg/ml uracil. Yeasts were grown with continuous shaking (200 rpm) at 37°C. Yeasts were grown to exponential phase for use in infection studies. For growth on solid medium, HMM was solidified with 0.6% agarose and supplemented with 25 μM FeSO_4_. Growth of yeasts in liquid culture was quantified by measurement of culture turbidity (optical density at 595 nm). Mycelia were grown statically at 25°C. Growth of mycelia was initiated by inoculating liquid media with yeasts at a density of 500 yeasts/ml and incubating the culture statically at 25°C for 11 days. Differentiation and growth of mycelia were scored by visual observation of hyphae formation at ×40 magnification.

10.1128/mBio.02712-19.6 TABLE S2 *Histoplasma* strains used in this study. The strains used included wild-type clinical isolates G217B, G186A, Hc01, WU24, Hc17, and Hc30. Mutant and transgenic strains were constructed in the G217B background. The table lists full genotypes as well as the relevant short designations (wild type [WT]). Depletion of gene function was achieved through RNAi or by one of the following gene mutations: deletion (Δ), T-DNA insertion, or CRISPR/Cas9-mediated coding sequence mutation. Download Table S2, PDF file, 0.1 MB. Copyright © 2020 Shen et al. 2020 Shen et al. This content is distributed under the terms of the Creative Commons Attribution 4.0 International license.

### Carbon substrates for *Histoplasma* growth.

To test the ability of *Histoplasma* to use different carbon substrates, *Histoplasma* yeasts and mycelia were grown in 3M media (48) with individual carbon substrates as the carbon source (cysteine was included as the organic sulfur source at 25 μM, a concentration insufficient for carbon needs for growth; see Fig. S1 in the supplemental material). Unless otherwise noted, the carbon substrates were prepared at 3% (wt/vol) and used at a final concentration of 1.5% (wt/vol). Carbon substrates included hexoses (glucose, mannose, fructose, and galactose), pentoses (ribose and xylose), disaccharides (sucrose, trehalose, and maltose), C2 or C3 carbon substrates (glycerol, pyruvate, lactate, and acetate), amino sugars (GlcNAc and NANA), and amino acids (Casamino Acids; Thermo Scientific). For tests of growth on exogenous saturated and unsaturated long-chain fatty acids, fatty acids (oleic, lineoleic, palmitoleic, myristic, stearic, and arachidonic acids) were added to media (final concentration, 0.1%) and the suspension was sonicated before addition to agarose. Short-chain fatty acids (formate, acetate, propionate, and butyrate) were added to liquid media at a 0.5% final concentration. For growth on protein and protein fragments, hemoglobin and gelatin were added to 3M media at a final concentration of 5 mg/ml. To generate proteolytic fragments, proteins (10 mg/ml) were digested with trypsin (in 5 mM Tris, pH 7.5), proteinase K (in 5 mM HEPES, pH 6.5), or cathepsin D (in 5 mM citrate, pH 3.5) for 24 h at 37°C. Digestion reaction mixtures were heated at 70°C for 1 h to inactivate the proteinase enzymes, and an equal volume of the digestion products was added to an equal volume of twice-concentrated 3M base medium without any carbon source. Yeasts were added to single-carbon media at 2 × 10^6^ yeasts/ml in 96-well microtiter plates and incubated at 37°C with twice-daily agitation (1,000 rpm for 60 s) (32) or spread on solid media.

### Macrophage cell culture.

The *lacZ*-expressing P388D1 cell line was created from the murine macrophage cell line P388D1 (ATCC CCL-46 [49]). *lacZ*-expressing P388D1 macrophage cells were maintained in Ham’s F-12 medium supplemented with 10% fetal bovine serum (FBS; Atlanta Biologicals). The cell line was cultured at 37°C in 5% CO_2_/95% air. For infection experiments, cells were cocultured with yeasts in Ham’s F-12 medium supplemented with 10% FBS.

Bone marrow cells were isolated from the femurs of C57BL/6 mice (Jackson Laboratory) and differentiated into macrophages (bone marrow-derived macrophages [BMDMs]) by culturing in RPMI 1640 supplemented with 10% FBS, 0.1% gentamicin sulfate, 5 μM 2-mercaptoethanol, and 10 ng/ml of mouse granulocyte-macrophage colony-stimulating factor (GM-CSF) for 7 days at 37°C in 5% CO_2_/95% air. Nonadherent cells were removed from tissue culture flasks by washing with phosphate-buffered saline (PBS).

### Isolation of *Histoplasma* mutants with attenuated intramacrophage growth.

*Histoplasma* strain WU15 or strain OSU233 was used as the genetic background for insertional mutagenesis. OSU233 was constructed to enable screening of intracellular *Histoplasma* growth using yeast-generated fluorescence (50). OSU233 was mutagenized by *Agrobacterium*-mediated transformation (17) using Agrobacterium tumefaciens strain LBA1100 harboring plasmid pBHt2 (51). Briefly, bacteria and yeasts were cocultured for 40 h on filters atop solid *Agrobacterium*-induction medium containing 0.1 mM acetosyringone at 25°C. The filters were then transferred to HMM containing 100 μg/ml uracil, 100 μg/ml hygromycin to select for *Histoplasma* transformants, and 10 μg/ml tetracycline to counterselect for A. tumefaciens. Individual transformants were picked into liquid HMM with 100 μg/ml uracil and used to infect confluent monolayers of P388D1 *lacZ*-expressing macrophage cells (49) in 96-well microtiter plates at an approximate multiplicity of infection (MOI) of 1:1 (yeasts/macrophages). Infected macrophages were grown in F-12 medium with 10% fetal bovine serum (FBS; Atlanta Biologicals) at 36°C in 5% CO_2_/95% air. Intramacrophage growth of OSU233 yeasts was monitored daily by measuring RFP fluorescence (530 nm excitation, 590 nm emission). After 7 days, surviving macrophages were quantified by determination of the remaining β-galactosidase activity (absorbance at 420 nm with correction at 595 nm) (49). Mutants with a reduction of at least 30% in intramacrophage yeast growth (red fluorescence) or a reduction of at least 30% in lysis of the macrophages were retained as candidate attenuated strains.

### Mapping and complementation of T-DNA insertional mutants at the *PCK1* locus.

The location of the T-DNA insertion in individual mutants was determined by thermal asymmetric interlaced PCR (TAIL-PCR [52]). A 100-ng volume of genomic DNA was used as the template for primary PCR, with a T-DNA left or right border-specific primer (LB11 or RB9, respectively) and one of four semirandom primers (LAD1 to LAD4). The primary PCR was diluted 500-fold and used as the template for the secondary PCR with nested left- or right-border primers (LB12 or RB10, respectively) and the AC1 primer. PCR products were sequenced and aligned to the *Histoplasma* genome sequence. Three mutants with insertions at the *PCK1* locus are reported in this study (OSU151, OSU304, and OSU305), and the mutant OSU304 was used as the representative Pck1-deficient strain in infection tests. The T-DNA insertions at the *PCK1* locus was confirmed by PCR and sequencing using *PCK1*-specific primers in conjunction with LB11 and RB9. Primer sequences are listed in the Table S3.

10.1128/mBio.02712-19.7 TABLE S3 Primers used in this study. Primer name and sequences (written 5′ to 3′) are given as well as the orientation of the primers relative to gene transcription (where applicable). Download Table S3, PDF file, 0.1 MB. Copyright © 2020 Shen et al. 2020 Shen et al. This content is distributed under the terms of the Creative Commons Attribution 4.0 International license.

To complement the *pck1* mutants, a 2.8-kb fragment consisting of the wild-type *PCK1* gene and 840 bp of upstream sequence was amplified by PCR from *Histoplasma* G217B genomic DNA using *PCK1*-specific primers and cloned into a *URA5*-based T-DNA plasmid fusing the *PCK1* gene with the sequence encoding a C-terminal FLAG epitope. Either the *PCK1* complementation vector (pCR646) or a control *gfp* expression vector (pCR628) was transformed by A. tumefaciens-mediated transformation into the *pck1* mutants.

### Depletion of metabolism gene functions by RNAi.

Metabolism gene functions were depleted from *Histoplasma* yeasts by RNA interference (RNAi [40]). The RNAi vector was created by PCR amplification of 500 to 700 nucleotides of the targeted gene coding region (coding DNA sequence [CDS]). Inverted copies of the target gene sequence were cloned into the pED02 *gfp* sentinel RNAi vector (38) by restriction enzyme-mediated directional cloning. For simultaneous depletion of hexokinase and glucokinase enzymes, a chimeric *HXK1*:*GLK1* molecule was created by overlap extension PCR (39) and cloned into the RNAi vector. RNAi vectors were transformed by *Agrobacterium*-mediated transformation into the GFP-expressing sentinel strain OSU194 (38). Ura-positive (Ura^+^) transformants were recovered, and the sentinel GFP gene fluorescence was quantified using a modified gel documentation system and ImageJ software (v1.44p; http://imagej.nih.gov/ij).

### CRISPR/Cas9-mediated *FBP1* gene knockout.

Mutation of the *FBP1* locus was done by CRISPR/Cas9-mediated gene editing using a modification of a Cas9 and CRISPR guide RNA (gRNA) expression plasmid (53). The Cas9 and gRNA expression cassettes from pPTS608-Cas9-hyg were moved to a telomeric *Histoplasma* vector with a *URA5* selection marker (pCR473 [54]). The gRNA protospacer sequence CGACGCGGGTGTGTCCGTCG targeting the *FBP1* gene was inserted into this vector to generate pCR724. WU15 was transformed with pCR724, and Ura^+^ transformants were passaged on HMM 3 times. Clonal populations were then analyzed using Tracking of Indels by DEcomposition (55; tide.deskgen.com) to identify populations with potential CRISPR/Cas9-mediated mutation of the locus. Isolates with indel mutations in the *FBP1* coding region were subjected to single-colony purification, sequenced to verify the mutation, and then passaged on media with uracil to cure the strain of the pCR724 CRISPR/Cas9 plasmid. A mutant harboring a +1 nucleotide insertion located 414 bp downstream of the start codon in the coding region was identified. To complement the *fbp1* mutant, a 1,168-bp fragment consisting of the wild-type *FBP1* gene was amplified by PCR from *Histoplasma* G217B genomic DNA and cloned into a *URA5*-based T-DNA plasmid fusing the *FBP1* coding sequence to sequence encoding a C-terminal FLAG epitope. This complementation plasmid (pQS79) was transformed into the *fbp1* mutant by A. tumefaciens-mediated transformation.

### Enzymatic activity assays.

All enzyme assays were performed at 37°C. One unit is equivalent to a 1 μM concentration of substrate converted per min. Protein concentrations were determined by the Bradford assay using bovine serum albumin as a standard.

Icl1 activity was measured as the production of a glyoxylate phenylhydrazone derivative (58). The reaction mixture (1.0 ml) consisted of 100 mM morpholinepropanesulfonic acid (MOPS)/KOH (pH 7.2), 5 mM MgCl_2_, 2 mM dithiothreitol, 3.5 mM phenylhydrazine, and *Histoplasma* cellular lysates (up to 200 μg protein). Assays were initiated by the addition of 2 mM isocitrate. The absorption was measured at 324 nm to determine the level of production of the glyoxylate phenylhydrazone derivative.

Fbp1 activity was measured as the production of NADPH using a coupled-enzyme assay (59). The reaction mixture (1.0 ml) consisted of 50 mM HEPES (pH 7.2), 0.2 mM fructose-1,6-bisphophate, 5 mM MgSO_4_, 40 mM (NH_4_)_2_SO_4_, 0.1 mM EDTA, 0.15 mM NADP^+^, 0.5 U of glucose-6-phosphate isomerase, 1 U of glucose-6-phophate dehydrogenase, and *Histoplasma* cellular lysates (up to 200 μg protein). The absorption was measured at 365 nm to determine the level of production of NADPH.

### Metabolic gene expression determination.

Metabolic gene transcriptional analyses were performed by quantitative reverse transcription-PCR (qRT-PCR). Genes involved in glycolysis (*GLK1*, *HXK1*, *PFK1*, and *PYK1*), gluconeogenesis (*PCK1* and *FBP1*), and utilization of fatty acids (*FOX1*, *ICL1*, and *MLS1*) were tested in this study. Wild-type yeasts were grown in 3M medium with 1.5% glucose or 3M medium with 2% Casamino Acids to the exponential-growth phase and collected by centrifugation (2,000 × *g* for 5 min). In addition, yeasts were collected from 2 × 10^6^ macrophages infected for 24 h (MOI 1:1). Infected macrophages were lysed with H_2_O, and the yeast was recovered from the lysate by centrifugation (2,000 × *g* for 5 min). Yeasts were resuspended in TRIzol (Life Technologies), and the RNA was extracted by mechanical disruption of yeasts with 0.5-mm-diameter glass beads followed by column purification of nucleic acids (Direct-zol; Zymo Research). Following DNA removal performed with DNase (Turbo; Invitrogen), RNA was subjected to reverse transcription with Maxima reverse transcriptase (Thermo Scientific) primed with random pentadecamers. Quantitative PCR was carried out using gene-specific primer pairs with SYBR green-based detection of product amplification (Bioline). Changes in individual metabolic gene transcript levels relative to actin (*ACT1*) were determined using the cycle threshold (ΔΔ*C_T_*) method (56) after normalization of cycle thresholds to that of the *TEF1* gene.

### Glycolysis capability (extracellular acidification) analysis.

The basal glycolysis capacity of yeasts recovered from glucose medium, Casamino Acids medium, or BMDMs was determined by measurement of the extracellular acidification rate (ECAR) using a Seahorse XF24 analyzer (Agilent Technologies Inc., Santa Cruz, CA). Wild-type yeasts were grown at 37°C for 48 h in HMM, followed by dilution (1:5) into 3M medium containing either 10 mM glucose or 10 mM Casamino Acids as the sole carbon source for 24 h. For analysis of intramacrophage yeasts, BMDM monolayers were established in 6-well plates with 3 × 10^6^ cells/well. BMDMs were infected with wild-type yeasts at an MOI of 1:2 and were incubated in RPMI 1640 with 10% FBS at 37°C in 5% CO_2_/95% air for 24 h. Macrophages were lysed in sterile water, intramacrophage yeasts were collected by centrifugation (400 × *g* for 5 min), and yeasts were washed three times in water. Yeasts collected from glucose medium, Casamino Acids medium, or BMDMs were enumerated, spun (300 × *g* for 5 min) onto a Seahorse culture plate (Agilent Technologies Inc., Santa Cruz, CA) precoated with poly-l-lysine (3 μg, 70 to 150 kDa) at a concentration of 4 × 10^6^ yeasts in 150 μl of water, and incubated at 37°C in sterile water with a low CO_2_ concentration for 1 h for equilibration. Immediately prior to analysis, glucose minimal medium was added to reach a final concentration of 10 mM glucose at pH 7.0 in a total volume of 500 μl. The extracellular acidification rate (ECAR) was measured at 44 min and 70 min. The glycolysis inhibitor 2-deoxy-d-glucose (2-DG) (Sigma-Aldrich, St. Louis, MO) (final concentration, 25 mM) was added to each well at 50 min. The reported ECAR data (determined by the glycolytic capacity of the yeasts) were calculated by subtracting the ECAR at 70 min (ECAR generated by nonglycolytic acidification, after 2-DG addition) from the ECAR at 44 min (total ECAR, before 2-DG addition).

### Intramacrophage proliferation of *Histoplasma* yeasts.

Macrophage monolayers were established in 96-well plates by seeding with 3 × 10^4^ P388D1 cells. Macrophages were infected with yeasts at an MOI of 1:2 and were incubated in Ham’s F-12 medium with 10% FBS at 37°C in 5% CO_2_/95% air. After 1 h, the medium was replaced to remove any remaining extracellular yeasts. Immediately or at 48 h postinfection, intracellular yeasts were quantified by removal of any extracellular yeasts with the culture supernatant followed by lysis of the macrophages with sterile H_2_O. Intracellular yeasts were enumerated by plating serial dilutions of the macrophage lysate on solid HMM to enumerate CFU. The fold change in CFU between 0 h and 48 h was determined.

### Murine model of pulmonary histoplasmosis.

Wild-type C57BL/6 mice were infected with wild-type, mutant, or complemented *Histoplasma* strains by intranasal delivery of approximately 2 × 10^4^ yeast cells. Actual numbers of yeasts delivered were determined by plating serial dilutions of the inocula on solid media for enumeration of CFU. At 8 days postinfection, mice were euthanized, lungs were collected and homogenized in HMM, and serial dilutions of the homogenates were plated on solid HMM to determine the fungal burden (CFU). For the determination of the time course of infection, wild-type C57BL/6 mice were infected with *Histoplasma* by intranasal delivery of approximately 2 × 10^4^ yeast cells consisting of equal numbers of wild-type yeast cells (GFP negative; OSU296) and Pck1-deficient yeast cells (GFP fluorescent; OSU306) or of Fbp1-expressing yeast cells (RFP fluorescent; OSU296) and Fbp1-deficient yeast cells (RFP negative; OSU368). Fungal burdens in the lungs of infected mice were enumerated at 2, 4, 6, and 8 days postinfection. Green fluorescence, red fluorescence, or lack of fluorescence of colonies was determined with a modified transilluminator and image capture system (57) to perform deconvolution of the lung fungal burdens of individual strains from the mixed population. Animal experiments were performed in compliance with the National Research Council’s Guide for the Care and Use of Laboratory Animals and were approved by the Institutional Animal Care and Use Committee (IACUC) at The Ohio State University (protocol 2007A0241).

### Statistical analyses.

Data were analyzed by Student's *t* test (Prism v8; GraphPad Software) or Kruskal-Wallis one-way analysis of variance (ANOVA) on ranks with Dunn’s pairwise multiple-comparison test for determination of statistically significant differences, which are indicated in graphs with asterisk symbols (*, *P < *0.05; **, *P < *0.01; ***, *P < *0.001).
