# Supplementary material for: Metabolism of Gluconeogenic Substrates by an Intracellular Fungal Pathogen Circumvents Nutritional Limitations within Macrophages
Source: mBio. 2020 Apr 7;11(2):e02712-19. doi: 10.1128/mBio.02712-19 (PMC7157778; doi:10.1128/mBio.02712-19)
Supplement: TABLE S1 [file mBio.02712-19-st001.pdf]

Table S1. Yeast growth[1] of phylogenetically distinct *Histoplasma* strains on carbon substrates

	G186A (Panama)	Hc01 (NAm2)	WU24 (NAm1)	Hc17 (NAm1)	Hc30 (LAm)
Glucose	+	+	+	+	+
Mannose	+	+	+	+	+
Fructose	+	+	+	+	+
Galactose	-	+/-	+/-	+/-	-
Ribose	-	-	-	-	-
Xylose	-	-	-	-	-
Sucrose	-	-	-	-	-
Trehalose	-	-	-	-	-
Maltose	-	-	-	-	-
Glycerol	-	-	-	-	-
Pyruvate	+	+	+	+	+
Lactate	+	+	+	+	+
Acetate	-	-	-	-	-
CAA[2]	+	+	+	+	+
NAG[3]	+	+	+	+	+

[1] growth of strains was scored using a qualitative scale:
"+" represents good growth
"+/-" represents weak growth and development of pseudohyphae
"-" represents no growth

[2] CAA: casamino acids

[3] NAG: N-acetyl glucosamine
