# Supplementary material for: Metabolism of Gluconeogenic Substrates by an Intracellular Fungal Pathogen Circumvents Nutritional Limitations within Macrophages
Source: mBio. 2020 Apr 7;11(2):e02712-19. doi: 10.1128/mBio.02712-19 (PMC7157778; doi:10.1128/mBio.02712-19)
Supplement: TABLE S2 [file mBio.02712-19-st002.pdf]

Table S2. *Histoplasma* strains

| strain | genotype[1] | designation |
|---|---|---|
| G217B[2] | wild type NAm2 isolate (ATCC 26032) | WT |
| G186A[3] | wild type Panama isolate (ATCC 26029) | WT |
| Hc01[4] | wild type NAm2 isolate | WT |
| WU24[5] | wild type NAm1 isolate | NAm1 |
| Hc17 | wild type NAm1 isolate | WT |
| Hc30 | wild type LAm isolate | WT |
| WU15 | *ura5-42Δ* | |
| OSU151 | *ura5-42Δ* *pck1-1*::T-DNA[*hph*] | *pck1* |
| OSU177 | *ura5-42Δ* *zzz*::pCR623[*URA5*, *gfp*] | *PCK1* |
| OSU194[6] | *ura5-42Δ* *zzz*::pAG21[*apt3*, *gfp*] | |
| OSU233 | *ura5-42Δ* *zzz*::p*QS*01[*apt3*, *rfp*] | WT |
| OSU241 | *ura5-42Δ* *pck1-1*::T-DNA[*hph*] *zzz*::pCR623[*URA5*, *gfp*] | *pck1* |
| OSU243 | *ura5-42Δ* *pck1-1*::T-DNA[*hph*] *zzz*::pCR646[*URA5*, $P_{PCK1}$-*PCK1*] | *pck1*/*PCK1* |
| OSU289 | *ura5-42Δ* *zzz*::pAG21[*apt3*, *gfp*] *zzz*::pED02[*URA5*, *gfp*-RNAi] | *gfp*-RNAi |
| OSU290 | *ura5-42Δ* *zzz*::pAG21[*apt3*, *gfp*] *zzz*::pED04[*URA5*, *gfp*:*ICL1*-RNAi] | *ICL1*-RNAi |
| OSU292 | *ura5-42Δ* *zzz*::pAG21[*apt3*, *gfp*] *zzz*::p*QS*18[*URA5*, *gfp*:*FOX1*-RNAi] | *FOX1*-RNAi |
| OSU296 | *ura5-42Δ* *zzz*::p*QS*01[*apt3*, *rfp*] *zzz*::pKG06[*URA5*] | *PCK1* |
| OSU304 | *ura5-42Δ* *zzz*::p*QS*01[*apt3*, *rfp*] *pck1*-2::T-DNA[*hph*] | *pck1* |
| OSU305 | *ura5-42Δ* *zzz*::p*QS*01[*apt3*, *rfp*] *pck1*-3::T-DNA[*hph*] | *pck1* |
| OSU306 | *ura5-42Δ* *zzz*::p*QS*01[*apt3*, *rfp*] *pck1*-2::T-DNA[*hph*] *zzz*::pCR623[*URA5*, *gfp*] | *pck1* |
| OSU307 | *ura5-42Δ* *zzz*::p*QS*01[*apt3*, *rfp*] *pck1*-2::T-DNA[*hph*] *zzz*::pCR646[*URA5*, $P_{PCK1}$-*PCK1*] | *pck1/PCK1* |
| OSU308 | *ura5-42Δ* *zzz*::p*QS*01[*apt3*, *rfp*] *pck1*-3::T-DNA[*hph*] *zzz*::pCR623[*URA5*, *gfp*] | *pck1* |
| OSU309 | *ura5-42Δ* *zzz*::p*QS*01[*apt3*, *rfp*] *pck1*-3::T-DNA[*hph*] *zzz*::pCR646[*URA5*, $P_{PCK1}$-*PCK1*] | *pck1*/*PCK1* |
| OSU359 | *ura5-42Δ* *zzz*::pAG21[*apt3*, *gfp*] *zzz*::p*QS*42[*URA5*, *gfp*:*PYK1*-RNAi] | *PYK1*-RNAi |
| OSU368 | *ura5-42Δ* *zzz*::pAG21[*apt3*, *gfp*] *zzz*::p*QS*59[*URA5*, *gfp*:*FBP1*-RNAi] | *FBP1*-RNAi |
| OSU378 | *ura5-42Δ* *zzz*::pAG21[*apt3*, *gfp*] *zzz*::p*QS*64[*URA5*, *gfp*:*HXK1*:*GLK1*-RNAi] | *HXK1*:*GLK1*-RNAi |
| OSU403 | *ura5-42Δ* *fbp1-1* | *fbp1* |
| OSU405 | *ura5-42Δ* *fbp1-1* *zzz:pCR628[URA5, gfp]* | *fbp1* |
| OSU406 | *ura5-42Δ* *fbp1-1* *zzz:pQS79[URA5, FBP1]* | *fbp1/FBP1* |

[1] gene designations
- *apt3*: aminoglycoside 3'phosphotransferase
- *FBP1*: fructose-1,6-bisphosphatase
- *FOX1*: fatty acyl-CoA oxidase
- *gfp*: green fluorescent protein (E*GFP*)
- *GLK1*: glucose kinase
- *hph*: hygromycin phosphotransferase
- *HXK1*: hexokinase
- *ICL1*: isocitrate lyase
- *PCK1*: phosphoenolpyruvate carboxykinase
- *PFK1*: phosphofructokinase
- *PYK1*: pyruvate kinase
- *rfp*: red fluorescent protein (td-Tomato)
- *URA5*: orotidine-5'-phosphate

[2] source: ATCC 26032
[3] source: ATCC 26029
[4] source: Holbrook ED, et al., 2014
[5] source: Marion CL, et al., 2006
[6] source: Garfoot AL, et al., 2016
