# Supplementary material for: Metabolism of Gluconeogenic Substrates by an Intracellular Fungal Pathogen Circumvents Nutritional Limitations within Macrophages
Source: mBio. 2020 Apr 7;11(2):e02712-19. doi: 10.1128/mBio.02712-19 (PMC7157778; doi:10.1128/mBio.02712-19)
Supplement: TABLE S3 [file mBio.02712-19-st003.pdf]

Table S3. Primers used in this study

Primer	Primer sequence (5' to 3')	Direction[1]
PCK1-2	CGACTAGTGTCAAGCCGAAGCCCCGTTCGT	Reverse
PCK1-3	GCCTAGGCTTCAAGCTGCAGACCAGAC	Forward
GDH2-2	CGACTAGTCTTCTGCTCTATGAGTTTTGCG	Reverse
GDH2-5	GCGAAGCTTCAACCGACGCTTTCCG	Forward
LB11	CCAAAATCCAGTACTAAAATCCAGATCCCCCGA	
LB12	CGGCGTTAATTCAGTACATTAAAAACGTCCGCA	
RB9	CCGCACCGATCGCCCTTCCCAACAG	
RB10	GCCTGAATGGCGAATGCTAGAGCAGCTTG	
LAD-1	ACGATGGACTCCAGAGCGGCCGCVNVNNNGGAA	
LAD-2	ACGATGGACTCCAGAGCGGCCGCBNBNNNGGTT	
LAD-3	ACGATGGACTCCAGAGCGGCCGCVVNVNNNCCAA	
LAD-4	ACGATGGACTCCAGAGCGGCCGCBDNBNNNCGGT	
AC1	ACGATGGACTCCAGAG	
GLK1-4	AGCAGGACGGAGCTACATTGCG	Forward
GLK1-5	TTGGATTGCGAGATGGAGAAGG	Reverse
HXK1-4	CTACAGAGATGGACCAAGGGCT	Forward
HXK1-5	CGTTGACGCCTGTACCGAAGAT	Reverse
PFK1-1	CATTGCGGGTGGCTGGCTATGA	Forward
PFK1-2	CACCTTCAGCAACGATAACGAT	Reverse
PYK1-1	ACTGAAGGGACGGAGTTGGTTA	Forward
PYK1-2	GCAGGGAGCTGTCGTCTATGAT	Reverse
FBP1-1	CAAACGGCTCCTCTAATCCGCC	Forward
FBP1-2	CGTCGGATGTAGTACGAGATGG	Reverse
FBP1-16	AGGCGCGCCATGGCCGCTTCAAACGGCTCCT	Forward
FBP1-17	CGACTAGTTTGGCATACTTCTGATGGAACG	Reverse
PCK1-6	GTAAAGGAGCCCTCGTCCGAGAA	Forward
PCK1-4	GACGCTGATTCTGTAGCGCTCG	Reverse
ICL1-9	CATATCGAGGATCAAGCTCCTG	Forward
ICL1-10	GGGGGTCGATAGTGGATGTGAT	Reverse
MLS1-1	TCTTGCTCAGGATTACATTGGC	Forward
MLS1-2	AATCCCATCGACCGCAGTTCAA	Reverse
FOX1-5	ATGGTCCTCATCCATTTGTCGT	Forward
FOX1-6	GGATCAACATGCGAGAACCGTG	Reverse
ACT1-14	CCTCTTCCAACCCAGTGTCCTA	Forward
ACT1-6	GGACTCGTCGTACTCCTGCTTCG	Reverse
TEF1-8	GCTCTGCTTGCTTTCACCCTTG	Forward
TEF1-9	TCTCCTTGTTCCAGCCCTTGT	Reverse

[1] Direction relative to gene transcription
